# N-terminal-pro-B-type-natriuretic peptide associated with 2-year mortality from both cardiovascular and non-cardiovascular origins in prevalent chronic hemodialysis patients

**DOI:** 10.1080/0886022X.2018.1437047

**Published:** 2018-02-18

**Authors:** Chihiro Kawagoe, Yuji Sato, Tatsunori Toida, Hideto Nakagawa, Yasuhiro Yamashita, Akihiro Fukuda, Shuji Iwatsubo, Shouichi Fujimoto

**Affiliations:** a Dialysis Division, University of Miyazaki Hospital, Miyazaki, Japan;; b Department of Hemovascular Medicine and Artificial Organs, Faculty of Medicine, University of Miyazaki, Miyazaki, Japan;; c Department of Internal Medicine, Division of Circulatory and Body Fluid Regulation, Faculty of Medicine, University of Miyazaki, Miyazaki, Japan

**Keywords:** N-terminal-pro-B-type-natriuretic peptide, prognosis, chronic dialysis, cardiovascular disease, infection, malignancy

## Abstract

N-terminal-pro-B-type-natriuretic peptide (NT-proBNP) was a predictive marker of cardiovascular disease (CVD)-related death in chronic dialysis patients. NT-proBNP was also correlated with markers of inflammation, malnutrition and protein-energy wasting. We hypothesized whether NT-proBNP was also associated with non-CVD death in chronic dialysis patients. A prospective observational study for incidence of death in chronic dialysis patients was conducted. Prevalent chronic dialysis patients (*n* = 1310) were enrolled and followed for 24 months. One hundred forty-four deaths were recorded. Area under the curve using ROC analysis for NT-proBNP showed: all causes of death (0.761), CVD-related (0.750), infection and malignancy-related (0.702) and others and unknown (0.745). After adjusting for age, sex, hemodialysis vintage, cardiothoracic ratio, mean pre-dialysis systolic blood pressure, dry weight and basal kidney disease, the hazard ratios (95% confidence intervals) per 1-log NT-proBNP calculated using multivariate Cox analysis were: all causes of death, 3.83 (2.51–5.85); CVD-related, 4.30 (2.12–8.75); infection and malignancy-related, 2.41 (1.17-4.93); and others and unknown origin, 5.63 (2.57–12.37). NT-proBNP was significantly associated not only with CVD-relate but also with non-CVD-related deaths in this population of prevalent chronic dialysis patients.

## Introduction

Numerous factors have been surveyed as predictors of mortality in hemodialysis patients. In cardiovascular disease (CVD), death from classical Framingham atherosclerotic risk factors, hypertension, dyslipidemia, smoking, glucose intolerance and left ventricular hypertrophy have not been thought sufficient to predict mortality [1]. Blood pressure variability [2,3], chronic kidney disease related to mineral bone diseases [4,5], inflammation [6,7] and volume status instability or volume overload [8,9] have been reported as additional risk factors for CVD death as well as all-cause mortality. Regardless of the markers surveyed, a single independent risk factor for all causes of death in hemodialysis patients remains to be found [1]. While CVD, infections and malignancies are important causes of death among hemodialysis patients, the predictive markers of morbidity and mortality from infectious diseases and malignancies have not been fully studied.

The N-terminal of the prohormone brain natriuretic peptide (NT-proBNP) is an N-terminal inactive protein that is cleaved from proBNP to release active peptide BNP. NT-proBNP is a biomarker of left ventricular stress in non-CKD patients [10]. It is also of value as an independent predictor of congestive heart failure [11]. Its use as a predictive biomarker has also been noted in CKD patients because it has been found to be markedly elevated, especially in hemodialysis patients [12] because NT-proBNP is degraded by the kidney, whose function is almost totally diminished in hemodialysis patients, and proBNP is also degraded by adipose tissue, which is sometimes much lower in hemodialysis patients [13]. NT-proBNP has also been reported as a marker of volume overload [14], coronary artery disease [15] and left ventricular dysfunction [16,17] in dialysis patients. In longitudinal studies, it was reported that NT-proBNP was a predictor for all-cause mortality [14,16,18] and CVD-related death [14]. However, the number of surveyed patients was relatively small and the causes of death were not examined. NT-proBNP has recently been reported to correlate with inflammatory markers (interleukin-6 and C-reactive protein) [19], a malnutrition marker (serum albumin) [14], and protein-energy-wasting syndrome [19]. From these studies, we hypothesized that NT-proBNP levels might be associated not only with CVD-related deaths but also other causes of death. To this end, we conducted a prospective cohort study examining the relationship between NT-proBNP levels and all causes of death, as well as with other causes of death in a large cohort of chronic hemodialysis patients.

## Methods

### Ethical considerations

This study was conducted in accordance with the principles contained in the Declaration of Helsinki, and was granted ethical approval by the University of Miyazaki Research Ethics Committee (No. 516). Informed consent was not obtained from participants because all data were anonymized.

### Study participants

We enrolled 1310 prevalent chronic hemodialysis patients (58.8% men, mean age 67.9 years, 112 months mean hemodialysis vintage) from 27 dialysis centers on December 31, 2010. Using the same cohort as we previously reported [20], however, there were some differences because of different study enrollment process. Exclusion criteria were: patients <3 months hemodialysis vintage, <18 years old, pregnant women, hospitalized patients and patients who did not wish to participate in the study. Participants were followed for 24 months. During the study period, 65 patients (5.0%) were transferred to other clinics or hospitals, or lost to follow-up.

### Data collection

Clinical data comprising age, sex, hemodialysis vintage, basal kidney disease, pre-dialysis blood pressure, dry weight, cardiothoracic ratio (CTR), cause of death, and transfer to other clinics or hospitals were collected. All data on each patient was reported by an attending physician. This information for each patient was added to the patient’s own check sheet and the attending physician checked new events monthly, then the information including a questionnaire was sent back to the University of Miyazaki as we previously reported [20]. Mean pre-dialysis blood pressure was calculated from three consecutive dialysis sessions. All data were collected and stored in the Dialysis Division of the University of Miyazaki Hospital.

Each cause of death was reported by attending physicians and reconfirmed with medical records, searched by YS and TT if necessary. Causes of death were defined as we previously reported [20]. CVD death was defined as death from ischemic or hemorrhagic stroke, acute myocardial infarction, any cause of congestive heart failure, or rupture of an aortic aneurysm. Stroke was diagnosed using typical physical findings and imaging examinations. Acute myocardial infarction was diagnosed using typical electrocardiogram findings or an elevation in myocardium-derived enzymes. Cardiac disease was determined based on a previous history of ischemic heart disease and/or congestive heart failure. Ischemic heart disease was defined as a previous history of being hospitalized or medicated with drugs for a diagnosis of angina pectoris and/or myocardial infarction. Congestive heart failure was confirmed using an echocardiogram as well as by the patient requiring hospitalization with symptoms of dyspnea or edema. In this study, sudden death was categorized as ‘others’ because we would like to check the influence of NT-proBNP on valid CVD mortality as real reasons of sudden death sometimes obscure.

### Measurements

NT-proBNP was measured using electrochemiluminescence immunoassays (SRL Inc. Tokyo, Japan) using serum drawn before the first dialysis session. Blood pressure was measured in the supine position by trained staff after at least 5-min rest. The CTR was calculated using a chest X-ray taken before the first dialysis session.

### Statistical analysis

All statistical analyses were performed with SPSS version 20.0 J software (IBM Corp., Armonk, NY, USA). Data are expressed as mean ± SD, except for raw NT-proBNP, which is expressed as median and 25th–75th percentile. Clinical parameters according to classification of NT-proBNP quartiles were compared using one-way analysis of variance or the Kruskal–Wallis test as appropriate. Categorical parameters were compared using the chi-square test. To examine the correlation between NT-proBNP and clinical parameters, the Spearman rank correlation test was used.

The diagnostic performance of NT-proBNP for mortality was assessed by analysis of the receiver operating characteristic (ROC) curve. The ROC curve was a plot of sensitivity vs. (1-specificity) for all possible cut-off values. To identify the optimal cut-off point, we chose the closest point to the left upper corner of the ROC curve [21]. Survival curves divided at the cut-off point were calculated using the Kaplan–Meier method.

We used univariable and multivariable Cox analyses to examine the independent association between the level of NT-proBNP and mortality. In the multivariable analysis, these associations were assessed with adjustments for age, sex, hemodialysis vintage, CTR, pre-dialysis systolic blood pressure, dry weight and basal kidney disease. *p* Values <.05 was considered statistically significant.

## Results

### Distribution of NT-proBNP

NT-proBNP data were normally distributed in logarithmic transformed data but not in raw data. The median value (25th–75th percentile) of the raw data were 4855 (2209–12358) ng/L, mean value ± SD of the digit number was 3.74 ± 0.54. These data were clearly elevated compared with the normal range (<125 ng/L) in patients without kidney function failure.

### Comparison of logarithmic NT-proBNP with other parameters at baseline

**Figure 1.**
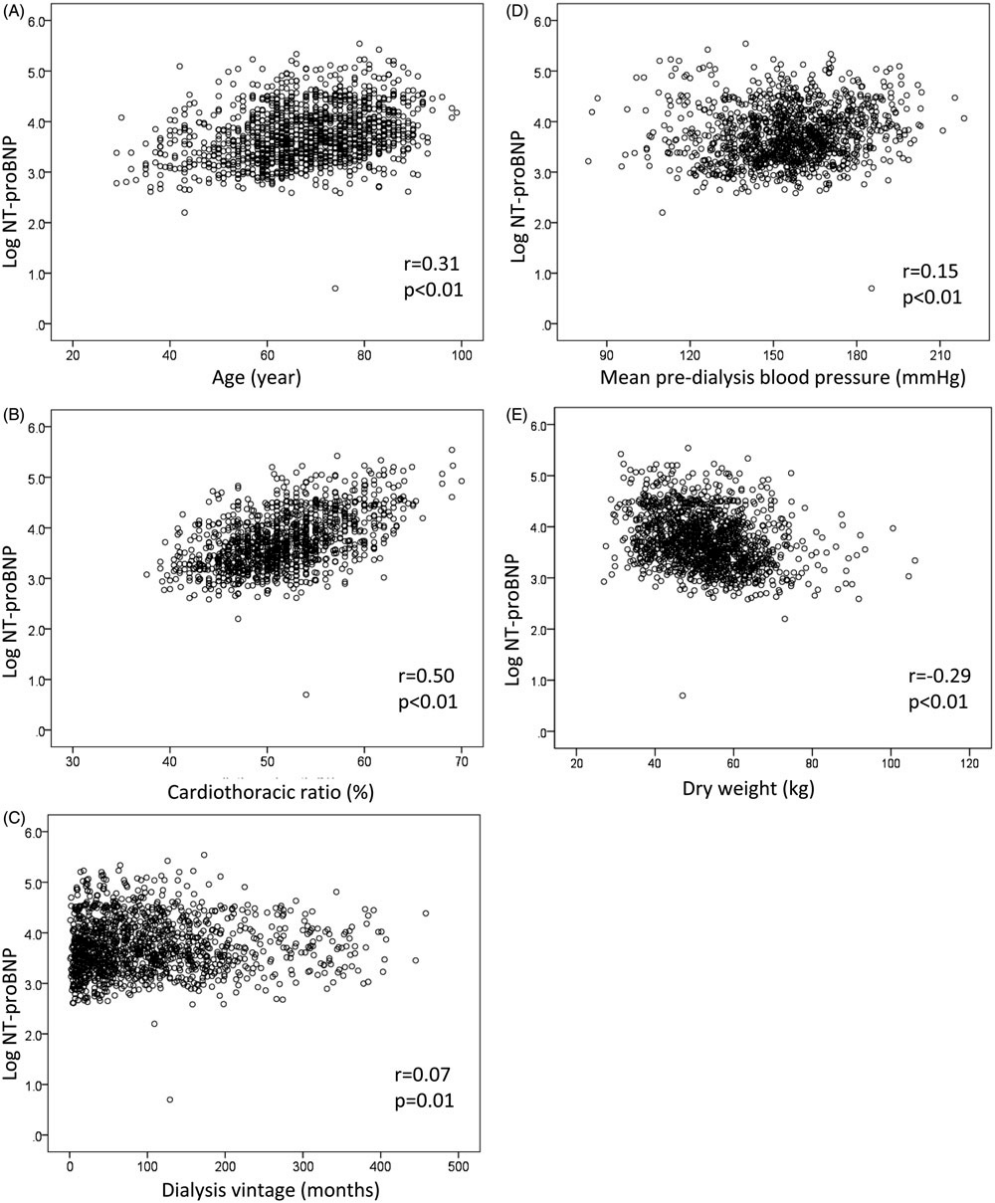
Comparison between logarithmic NT-proBNP and other clinical parameters at baseline. Logarithmic NT-proBNP correlated with age (*r* = 0.31, *p* < .01) **(**A**)** and cardiothoracic ratio (*r* = 0.50, *p* < .01) **(**B**)**, but not with hemodialysis vintage (*r* = 0.07, *p* = .012) **(**C**)**, mean pre-dialysis systolic blood pressure (*r* = 0.15, *p* < .01) **(**D**)**, or dry weight (*r*=-0.29, *p* < .01) **(**E**)**. All data were extracted using Spearman’s rank correlation tests.

Logarithmic NT-proBNP correlated well with age (*r* = 0.31, *p* < .01) (Figure 1(A)) and CTR (*r* = 0.50, *p* < .01) (Figure 1(B)), but not with hemodialysis vintage (*r* = 0.07, *p* = .01) (Figure 1(C)), mean pre-dialysis systolic blood pressure (*r* = 0.15, *p* < .01) (Figure 1(D)), or dry weight (*r* = −0.29, *p* < .01) (Figure 1(E)).

### Causes of death

During the study period, a total of 144 deaths were recorded (Table 1). Among them, 54 were of cardiovascular origin, 33 were of infectious origin, 19 were from malignancies, and 38 were from other or unknown reasons.

**Table 1.** Causes of death.

Causes of death	*n*	Subtotal
CVD		
Congestive heart failure	29	
Acute myocardial infarction	9	
Brain hemorrhage	8	
Brain infarction	6	
Rupture of aortic aneurysm	2	54
Infection		
Pneumonia	21	
Sepsis	10	
Others	2	33
Malignancy		
Lung cancer	5	
Gastric cancer	3	
Colon cancer	3	
Hepatocellular carcinoma	2	
Others	6	19
Others		
Sudden death	9	
Senile deterioration	4	
Respiratory failure	3	
Gastrointestinal bleeding	2	
Ileus	2	
Unknown	18	38
	Total	144

CVD: cardiovascular disease.

### Characteristics of participants

When the clinical data were compared among survivors and non-survivors, who were classified into four causes of death, non-survivors were found to be significantly older than survivors (Table 2). NT-proBNP, basal kidney disease, hemodialysis vintage, dry weight, pre-dialysis systolic blood pressure and CTR were significantly different between groups.

**Table 2.** Comparison between survivors and non-survivors.

		Non-survivors				
	Survivors	CVD	Infection	Malignancy	Others/unknown	*p*-value
*n*	1166	54	33	19	38	
Age (years)	66.7 (12.3)	75.6 (11.7)	79.4 (8.8)	75.5 (12.5)	77.1 (10.7)	<.001
Sex, men (%)	58.8%	53.7%	63.6%	68.4%	47.4%	.458
NT_proBNP (ng/L)	4200 (2030–10535)	13800 (6203–34875)	16604 (5686–31707)	8970 (7224–13376)	17147 (7726–54123)	<.001
Log NT_proBNP	3.68 (0.52)	4.21 (0.50)	4.13 (0.47)	3.96 (0.47)	4.31 (0.53)	<.001
Basal kidney disease						<.001
Diabetes mellitus	23.1%	25.9%	24.2%	31.6%	36.8%	
Glomerulonephritis	44.4%	33.3%	21.2%	31.6%	21.1%	
Hypertension	12.3%	25.9%	36.4%	26.3%	23.7%	
Polycystic kidney	4.8%	0%	6.1%	5.3%	0%	
Others/unknown	15.4%	14.8%	12.1%	5.3%	18.4%	
Hemodialysis vintage (months)	103.5 (90.0)	99.7 (83.8)	101.3 (93.5)	43.6 (39.5)	69.5 (51.2)	.009
Dry weight (kg)	53.6 (11.0)	48.8 (10.3)	45.9 (8.3)	49.5 (9.5)	45.7 (11.6)	<.001
Pre-systolic blood pressure (mmHg)	156 (19)	148 (27)	148 (22)	156 (20)	154 (24)	.020
CTR (%)	51.3 (5.2)	56.5 (5.5)	54.8 (4.9)	50.7 (5.6)	55.9 (5.8)	<.001

Continuous parameters are expressed as mean (SD) and are compared using one-way ANOVA, and categorical parameters are compared using the chi-squared test. Raw NT-proBNP data are expressed as medians and 25th–75th percentile and compared using the Kruskal–Wallis test. CVD: cardiovascular disease; CTR: cardiothoracic ratio.

### Prediction of mortality

**Figure 2.**
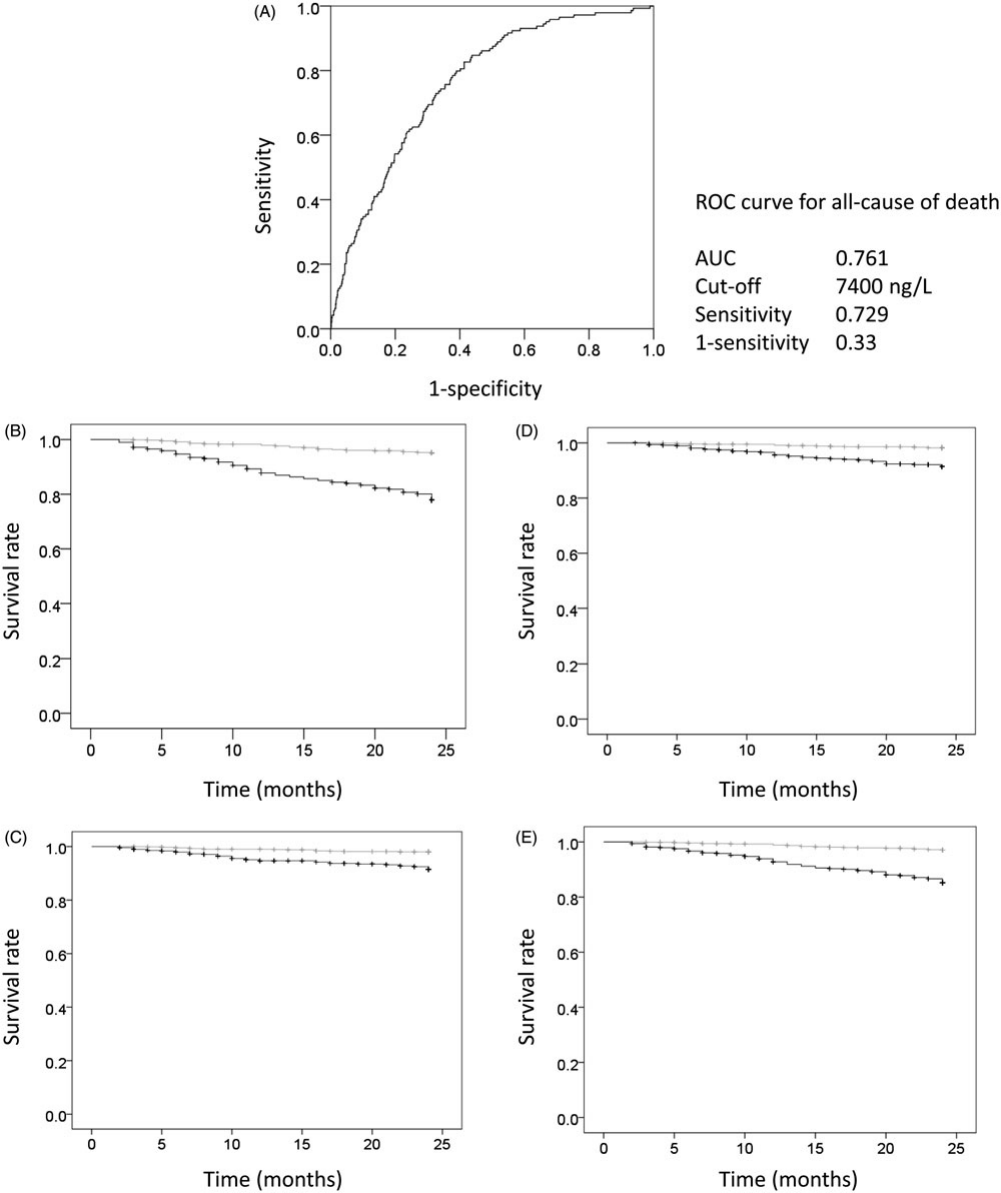
Receiver operating characteristic (ROC) curve for all causes of death and Kaplan–Meyer analysis. Sensitivity and specificity for all causes of death was assessed using the ROC curve (A). For the two-year all-cause mortality prediction, the area under the curve (AUC) was 0.761 and the cut-off point was 7400 ng/L. AUC for other causes of death were: 0.750 for CVD-related death, 0.702 for infection and malignancy-related death, and 0.745 for others and unknown cause of death. Using the cut-off point for all causes of death, the mortality rate was clearly and significantly divided into better and worse prognoses using the Kaplan–Meier method (all causes of death (B), cardiovascular-related death (C), infection and malignancy-related death (D), and others and unknown cause of death (E)). Gray line, NT-proBNP <7400 ng/L; black line, NT-proBNP ≥7400 ng/L.

To clarify the cut-off point that would distinguish between a better and worse prognosis, ROC curves and Kaplan–Meier analyses were performed. Sensitivity and specificity for all causes of death was assessed using ROC (Figure 2(A)). For the 2-year all-cause mortality prediction, the area under the curve (AUC) was 0.761 and the cut-off point was 7400 ng/L. AUC of other causes of death were: 0.750 for CVD-related death; 0.702 for infection and malignancy-related death; and 0.745 for others and unknown cause of death. Using the cut-off point of all causes of death, the mortality rate was clearly and significantly divided into better and worse prognoses using the Kaplan–Meier method (all causes of death, Figure 2(B); cardiovascular-related death, Figure 2(C); infection and malignancy-related death, Figure 2(D); others and unknown cause of death, Figure 2(E)).

We examined the significance of NT-proBNP at predicting mortality using Cox analysis (Table 3). With univariate analysis, log NT-proBNP was significantly associated with not only all causes of death but also CVD-related, infection and malignancy-related, and others and unknown cause of death. With adjustments for age, sex, hemodialysis vintage, CTR, mean pre-dialysis systolic blood pressure, dry weight, and basal kidney disease, in addition to an older age, log NT-proBNP was still a significant predictor for both CVD related, infection and malignancy-related, and others and unknown cause of death. The hazard ratios (95% confidence intervals) per 1-log NT-proBNP calculated using multivariate Cox analysis were: all causes of death, 3.83 (2.51–5.85); CVD-related, 4.30 (2.12–8.75); infection and malignancy-related, 2.41 (1.17–4.93); and others and unknown cause of death, 5.63 (2.57–12.37).

**Table 3.** Univariate and multivariate cox analysis for each cause of death.

		Causes of death			
	Covariates	All causes	CVD	Infection/Malignancy	Others/Unknown
Univariate	LogNT-proBNP, +1	**4.62 (3.48–6.14)**	**4.95 (3.11–7.89)**	**3.26 (2.03–5.25)**	**6.74 (3.87–11.76)**
Multivariate	LogNT-proBNP, +1	**3.83 (2.51–5.85)**	**4.30 (2.12–8.75)**	**2.41 (1.17–4.93)**	**5.63 (2.57–12.37)**
	Age, +5 y	**1.30 (1.17–1.44)**	**1.32 (1.11–1.57)**	**1.37 (1.14–1.64)**	**1.22 (1.01–1.47)**
	Sex, men vs. women	**1.65 (1.03–2.64)**	1.04 (0.49–2.21)	**3.24 (1.40–7.51)**	1.40 (0.58–3.37)
	Hemodialysis vintage, +1 month	1.00 (1.00–1.00)	1.00 (1.00–1.01)	1.00 (0.99–1.00)	1.00 (0.99–1.00)
	CTR, +5%	1.02 (0.83–1.25)	1.16 (0.82–1.65)	0.91 (0.64–1.29)	0.97 (0.66–1.43)
	pre-dialysis SBP, +10 mmHg	**0.88 (0.81–0.97)**	**0.81 (0.70–0.94)**	0.93 (0.79–1.08)	0.94 (0.80–1.10)
	Dry weight, +5 kg	**0.86 (0.75–0.98)**	1.09 (0.89–1.33)	**0.70 (0.55–0.89)**	**0.75 (0.58–0.98)**
	BKD, diabetes mellitus vs. others	1.38 (0.89–2.12)	1.19 (0.57–2.49)	1.59 (0.77–3.32)	1.38 (0.64–3.00)

Data are expressed as hazard ratios (95% confidence intervals). Bold letters indicate a significant hazard ratio. CVD: cardiovascular disease; CTR: cardiothoracic ratio; SBP: systolic blood pressure; BKD: basal kidney disease.

## Discussion

We identified that NT-proBNP was associated with future infection and malignancy-related or others and unknown cause of death in addition to CVD-related deaths in this population of prevalent chronic dialysis patients.

NT-proBNP accumulates in patients with kidney failure, especially those on chronic dialysis. Our data revealed that the accumulated NT-proBNP level was strongly associated with not only CVD-related deaths but also non-CVD-related ones. Recently, increasing NT-proBNP levels during the study period were reported to be associated with sudden death, cardiovascular events, and mortality in diabetic dialysis patients [22]. NT-proBNP was measured only once, then the participants were followed for a relatively short period (two years), so we could observe the effects of baseline NT-proBNP levels on mortality.

NT-proBNP has been reported as a predictor for cardiovascular mortality in dialysis patients [14]. Furthermore, because NT-proBNP has been reported to be a marker of volume overload [23] and left ventricular systolic dysfunction [16–18,24] in dialysis patients, it is possible that NT-proBNP is a predictive marker. BNP and NT-proBNP levels have been shown to be superior to atrial natriuretic peptide levels for the diagnosis of the presence, severity and prognosis of heart failure in non-CKD patients [25]. In older aged cohorts, however, BNP and NT-proBNP levels for the prediction of heart failure has not been established in the general population [26]. The cohort in the current study also comprised of older participants, and increments in age were significant factors for each cause of death. It is noteworthy that NT-proBNP was still an independent and significant associated factor not only for CVD-related death but also for other causes of death.

The reasons that NT-proBNP, considered a circulatory marker, was associated with non-CVD-related death in this study are not known. We can speculate that those patients might have had chronic heart failure resulting in a chronic pulmonary congestive state, regardless of severity. Such a state is a known risk factor for morbidity from pneumonia [27,28] and mortality from pneumonia [29]. Pneumonia was a dominant cause (over 60%) of infection in our cohort. Recent studies have reported that NT-proBNP positively correlated with interleukin-6 and C-reactive protein [19] and inversely correlated with serum albumin [14], so that NT-proBNP was considered a marker of inflammation or malnutrition. Also, the receptor for natriuretic peptide A and B (natriuretic peptide receptor A, NPRA) has been reported to be involved in immune and inflammatory reactions [30] as well as in tumor growth [31]. Furthermore, BNP enhanced vascular permeability [32] via morphological vascular endothelial cell changes. These are other possible explanations for why NT-proBNP may be associated with future non-CVD deaths.

ProBNP was also degraded by adipose tissue, which is sometimes much less in dialysis patients, especially those with a lower body mass index (BMI) [13]. These patients presented with malnutrition, inflammation and higher proBNP levels concurrently. However, even though we do not have BMI data, patients with a lower BMI could have developed an infection.

The reasons why higher NT-proBNP levels are associated with malignancy-related death are unclear; however, some of these might be as follows. First, malignant patients usually have a lower BMI, and as stated above, these patients have been found to have higher levels of proBNP [13]. Second, a positive correlation between BNP and C-reactive protein was observed in cancer patients without heart failure [33]. Altogether, patients with malnutrition and in an inflammatory state showed higher proBNP levels because of reduced degradation by adipose tissue [13] and up-regulation of BNP at the transcriptional and translational levels, respectively [34].

In multivariate analysis, increments in pre-dialysis systolic blood pressure were associated with a better prognosis, especially for all causes and cardiovascular-related deaths. In the general population, controlling and lowering blood pressure is recommended to prevent heart failure; however, for dialysis patients, there is no accurate optimum blood pressure level. At times, physicians have observed that an increased dialysis vintage results in lowered blood pressure, yet a lower blood pressure does not always mean a good prognosis.

This study had some limitations. The cohort comprised of prevalent chronic hemodialysis patients, and was not an incident cohort. Therefore, some sick patients might have died before being enrolled in our study. We could not identify the real cause of death among sudden death or unknown origin cases. Among them, there might have been some patients who had suffered from acute myocardial infarction or stroke. We did not have echocardiogram data, which have been reported to show a significant relationship with NT-proBNP [17,18,24]. In addition, laboratory data were lacking, as serum albumin and C-reactive protein have been reported to be strong significant predictors of mortality [7,8,35]. BMI data, which have been reported to be negatively correlated with NT-proBNP [36], were also lacking.
